# Estradiol (E2) Improves Glucose-Stimulated Insulin Secretion and Stabilizes GDM Progression in a Prediabetic Mouse Model

**DOI:** 10.3390/ijms23126693

**Published:** 2022-06-15

**Authors:** Moritz Liebmann, Melissa Asuaje Pfeifer, Katharina Grupe, Stephan Scherneck

**Affiliations:** Institute of Pharmacology, Toxicology and Clinical Pharmacy, Technische Universität Braunschweig, Mendelssohnstraße 1, D-38106 Braunschweig, Germany; m.liebmann@tu-braunschweig.de (M.L.); melissa.asuaje-pfeifer@tu-braunschweig.de (M.A.P.); k.grupe@tu-braunschweig.de (K.G.)

**Keywords:** estradiol, prediabetes, gestational diabetes, liver, pancreas, multi-organ disease

## Abstract

Female New Zealand obese (NZO) mice are an established model of preconceptional (pc.) prediabetes that progresses as gestational diabetes mellitus (GDM) during gestation. It is known that NZO mice show improvement in insulin sensitivity and glucose-stimulated insulin secretion (GSIS) during gestation in vivo. The latter is no longer detectable in ex vivo perifusion experiments in isolated islets of Langerhans, suggesting a modulation by extrapancreatic factors. Here, we demonstrated that plasma 17β-estradiol (E2) levels increased markedly in NZO mice during gestation. The aim of this work was to determine whether these increased E2 levels are responsible for the improvement in metabolism during gestation. To achieve this goal, we examined its effects in isolated islets and primary hepatocytes of both NZO and metabolically healthy NMRI mice. E2 increased GSIS in the islets of both strains significantly. Hepatic glucose production (HGP) failed to be decreased by insulin in NZO hepatocytes but was reduced by E2 in both strains. Hepatocytes of pregnant NZO mice showed significantly lower glucose uptake (HGU) compared with NMRI controls, whereby E2 stimulation diminished this difference. Hepatocytes of pregnant NZO showed reduced glycogen content, increased cyclic adenosine monophosphate (cAMP) levels, and reduced AKT activation. These differences were abolished after E2 stimulation. In conclusion, our data indicate that E2 stabilizes and prevents deterioration of the metabolic state of the prediabetic NZO mice. E2 particularly increases GSIS and improves hepatic glucose utilization to a lower extent.

## 1. Introduction

It is known that differences in the manifestation and thus also in the pathophysiology of type 2 diabetes (T2DM) exist between males and females. Male patients with T2DM have a higher risk of developing microvascular complications, whereas females have a higher morbidity in cardiovascular disease [1]. However, the physiological differences between the sexes, which differ in the extent of insulin resistance (IR), body fat distribution, and energy balance, are important for the understanding of the diseases’ aetiology [2]. Considering these differences between males and females, the gonadal steroids are of significant importance as they regulate both the reproductive system and metabolism [3]. Therefore, endocrine imbalances may have severe consequences. Estrogens belong to the sex steroid hormones and are derived from androgenic precursors, whereas E2 and its metabolite estrone (E1) can be converted to estriol (E3). However, E2 is the most bioactive endogenous estrogen, as it has about 10 times the potency of E1 and 100 times the potency of E3 [4,5,6]. The Women’s Health Across the Nation (SWAN) study proposed that reduced E2 levels lead to a 47% higher risk of T2DM during the menopausal transition, and even lower premenopausal E2 levels were associated with a higher diabetes risk [7]. The strong correlation between E2-deficiency and adverse metabolic outcomes has been observed in humans, particularly in the field of hormone replacement therapy (HRT) [8]. Clinical trials with postmenopausal patients showed that HRT was associated with an improvement of IR and a decrease in plasma glucose levels [9]. Overall, there is evidence which suggests a protective E2 effect on glycemic control in humans [10]. Besides the ovaries, which are the site of E2 synthesis for maintaining reproductive processes, hepatic tissue expresses ERα, and is one of the most inducible in vivo targets of E2 [11,12]. E2 has been described to have a modulatory effect on non-alcoholic fatty liver disease (NAFLD). Thus, long-term treatment with the selective estrogen receptor modulator (SERM) Tamoxifen was shown to significantly increase the risk of fatty liver development in patients with breast cancer. SERMs may attenuate fatty acid oxidation by blocking the ERs, thereby acting as potent inhibitors of estrogen signaling [13]. Furthermore, ERα-mediated signaling in non-hepatic tissues has been shown to be essential for the protection against high-fat diet (HFD)-induced steatosis [14]. Mice lacking E2 synthesis due to ovariectomy develop a large increase in liver fat, although this effect can be reversed by the administration of E2 [15]. This provides conclusive evidence for the major influence of E2 on glucose homeostasis through endocrine mediation of insulin secretion and insulin sensitivity.

Therefore, E2 might be an important factor for glucose metabolism in the context of GDM. The global prevalence of GDM is estimated at 14% by the International Diabetes Federation, which illustrates the enormous impact of this disease considering associated health consequences for mother and offspring [16]. The high incidence, with 55% and 6% of women developing prediabetes and T2DM after a previous GDM diagnosis, highlights the future economic burden on healthcare systems worldwide [17].

The E2 level gradually increases during pregnancy in humans and rodents [18,19]. It is acknowledged to play an important role in triggering ovulation before conception, but its actions during pregnancy are not fully understood. Data gathered in baboons suggest that E2 is essential for the maintenance of pregnancy, as treatment with an aromatase inhibitor results in lower maintenance rates and delivered live fetuses or neonates compared to untreated controls [20]. Moreover, an enhancing effect on GSIS has been described during healthy pregnancy [21,22]. However, studies conducted in GDM patients are conflicting. It has been reported that E2 plasma levels during gestation are lower overall in diabetic patients and women with preconceptional diabetes reveal the lowest levels during the first and second trimester compared with healthy women [23,24]. Nevertheless, it should be noted that in other GDM cohorts, E2 plasma and serum levels tended to be higher compared to healthy controls, yet in further cohorts it was reported that E2 serum levels did not differ between patients and controls [25,26,27]. This could be explained via an overstimulation of ERα by E2, which could provoke IR in the liver and depletion of β-cells, possibly contributing to diabetic phenotypes [28]. Moreover, GDM was associated with elevated E2 cord plasma concentrations in male newborns [29].

NZO mice are a polygenic model with a long history in the field of T2DM research and various gene-gene interactions are reflective of human pathophysiology [30]. Interestingly, due to the protective effect of endogenous E2 in females, only male mice are affected by T2DM [31]. We have recently shown that female NZO mice are a suitable model for GDM research [32]. We demonstrated that IGT is already impaired preconceptionally and persists during gestation. This form of IGT does not worsen but improves slightly during gestation. However, the improved stimulability of insulin secretion detected in vivo was not sufficient to reverse IGT in NZO mice. This was demonstrated by an improvement of whole-body insulin sensitivity and GSIS in vivo. In addition, we found that preconceptionally elevated glucagon levels stabilized slightly. Interestingly, the enhancement of GSIS is not detectable in ex vivo perifusion experiments. This suggests that pregnancy-associated extrapancreatic modulators are responsible for the enhancement of GSIS [32]. Therefore, the aim of this study was to determine whether E2 during gestation in NZO mice is responsible for stabilizing IGT rather than exacerbating it. For this purpose, isolated islets of Langerhans from NZO mice were stimulated with E2 to test for the improvement of GSIS and possible influences on glucagon secretion. Furthermore, we investigated the influence of E2 on hepatic glucose metabolism and signaling by determination of insulin induced protein kinase B (PKB/AKT) activation as an indicator for improved insulin sensitivity. Therefore, primary hepatocytes were isolated, cultured, and stimulated accordingly.

## 2. Results

### 2.1. Increased Plasma E2 Levels in NZO Mice during Gestation

**Figure 1:**
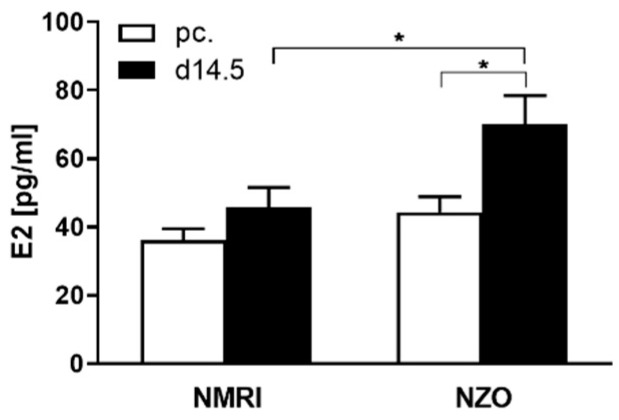
Elevated E2 levels in NZO mice during gestation. Plasma E2 concentrations in NMRI and NZO mice fed ad libitum at time points preconceptional (white bars) and d14.5 (black bars). Data are presented as means ± SEM (n = 6 animals per group). * *p* < 0.05.

Plasma E2 levels in both strains at two time points: preconceptional and at day 14.5 of gestation (d14.5), were determined. Preconceptionally, the E2 levels did not differ between NZO and NMRI controls. At time point d14.5 both strains revealed an increment in E2 levels compared to preconceptional, albeit a significant increase was observed solely in the NZO mice (70.07 ± 8.40 vs. 44.33 ± 4.56 pg/mL, *p* < 0.05). As a consequence, NZO mice showed a significant 1.5-fold increase in E2 levels during pregnancy compared to the NMRI controls (NZO vs. NMRI: 70.07 ± 8.40 vs. 45.81 ± 5.72 pg/mL, *p* < 0.05) (Figure 1).

### 2.2. Effects of E2 on Insulin and Glucagon Secretion in Freshly Isolated Islets

**Figure 2:**
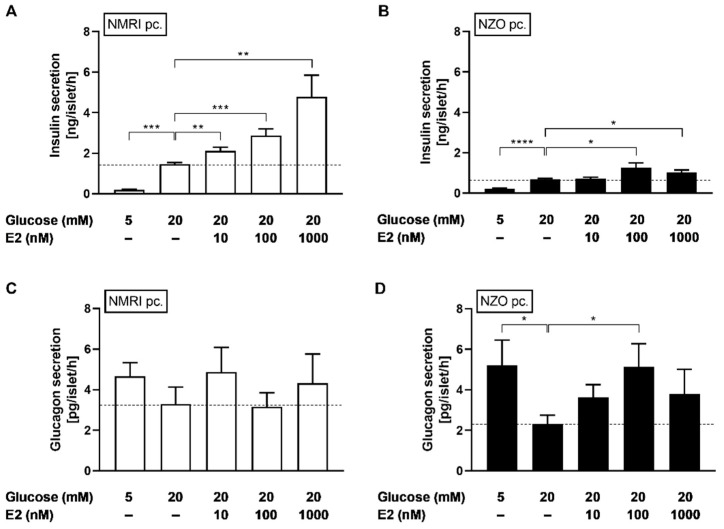
E2 improved insulin secretion but not glucagon secretion in freshly isolated islets. Insulin secretion (upper graphs) and glucagon secretion (lower graphs) of (**A**,**C**) preconceptional NMRI and (**B**,**D**) NZO islets after static incubation with 5 or 20 mM glucose in combination with E2. Data are presented as means ± SEM ((**A**) n = 7–9, (**B**) n = 8–10, (**C**) n = 8–9 and (**D**) n = 9–10 animals per group). * *p* < 0.05; ** *p* < 0.01; *** *p* < 0.001, **** *p* < 0.0001.

To investigate whether improved GSIS in vivo is linked to increased plasma E2 levels during gestation in NZO mice, preconceptional islets were induced to a gestational-like state by stimulation with E2. Insulin secretion of isolated islets was enhanced in a dose-dependent manner by 10, 100, and 1000 nM E2 in NMRI control mice (10/100/1000 nM E2 + 20 mM glucose vs. 20 mM glucose: 2.12 ± 0.18/2.87 ± 0.33/4.79 ± 1.06 vs. 1.46 ± 0.08 ng/islet/h, *p* < 0.01/0.001/0.01) (Figure 2A) and by 100 as well as 1000 nM E2 in NZO mice (100/1000 nM E2 + 20 mM glucose vs. 20 mM glucose: 1.26 ± 0.23/1.02 ± 0.13 vs. 0.68 ± 0.05 ng/islet/h, *p* < 0.05/0.05) (Figure 2B). E2 showed no significant effect on glucagon secretion in NMRI islets (Figure 2C), but there was an increase of glucagon secretion after stimulation with 100 nM E2 in NZO islets (100 nM E2 + 20 mM glucose vs. 20 mM glucose: 5.13 ± 1.14 vs. 2.33 ± 0.42 pg/islet/h, *p* < 0.05) (Figure 2D). Incubation with E2 resulted in an increase in GSIS in both strains and enhanced glucagon secretion in NZO mice.

### 2.3. Effects of E2 on Hepatic Glucose Utilization in Primary Hepatocytes

**Figure 3:**
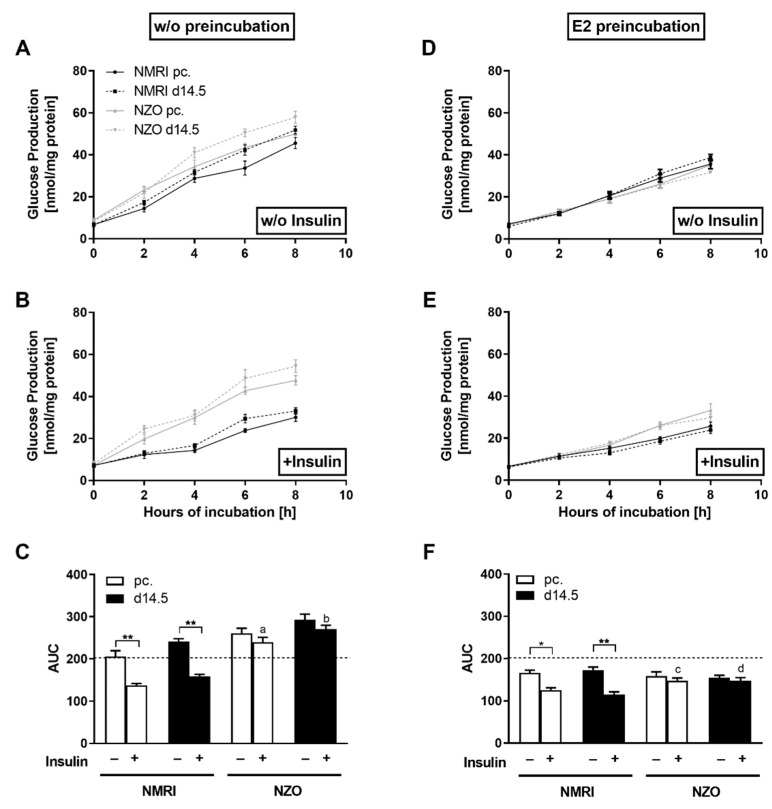
Altered glucose utilization in primary NZO hepatocytes and the effect of E2. Glucose production in cultured primary hepatocytes of NMRI and NZO mice at time points preconceptional and d14.5. (**A**,**B**) Preincubation without (*w*/*o*) (**D**,**E**) or with E2 and (**A**,**D**) stimulation *w*/*o* or (**B**,**E**) with insulin. Area under the curve (AUC) for glucose production using the trapezoidal rule at time points preconceptional (white bars) and d14.5 (black bars). (**C**) *w*/*o* and (**F**) after preincubation with E2. Data are presented as means ± SEM (n = 5 animals per group). * *p* < 0.05, ** *p* < 0.01, NMRI pc. *w*/*o* vs. NZO pc. *w*/*o*: a *p* < 0.05, NMRI d14.5 *w*/*o* vs. NZO d14.5 *w*/*o*: b *p* < 0.05, NMRI pc. + E2 vs. NZO pc. + E2: c *p* < 0.05, NMRI d14.5 + E2 vs. NZO d14.5 + E2: d *p* < 0.05.

Based on the increased adaptation of NZO mice in plasma E2 concomitant with E2-induced enhanced insulin secretion in freshly isolated islets, the possible effects of E2 on hepatic glucose metabolism were investigated. Compared to NMRI, primary NZO cells produced significant higher amounts of glucose both preconceptionally and at d14.5 (Figure 3A). When stimulated with insulin, hepatic HGP was significantly suppressed in NMRI hepatocytes, preconceptionally this effect was stronger due to the pregnancy-induced IR at d14.5 (Figure 3B). In contrast, NZO cells showed a significantly reduced response to insulin as a stimulant, resulting in a nearly absent reduction in HGP in the NZO cells after insulin stimulation. Therefore, the NZO cells displayed a diminished response to insulin, while the NMRI controls responded at both time points with a significant decrease in HGP (Figure 3C). After preincubation with E2, there was a marked response to the stimulant with reduced HGP in the cells of both strains. Consequently, there were no differences between the NZO and NMRI cells at either time point (NZO vs. NMRI, preconceptional: 158.72 ± 9.80 vs. 166.14 ± 6.47, n.s.; NZO vs. NMRI, d14.5: 154.43 ± 5.89 vs. 172.38 ± 7.57 nmol/mg protein × 8 h, n.s.) (Figure 3D). In co-stimulation with insulin, E2 significantly enhanced the HGP in the NMRI controls, whereas no further reduction was seen in NZO cells (Figure 3E,F). Thus, the HGP was significantly increased in NZO cells at both time points after co-stimulation of E2 with insulin compared to the NMRI controls (NZO vs. NMRI, preconceptional: 148.25 ± 5.52 vs. 125.75 ± 5.35, *p* < 0.05; NZO vs. NMRI, d14.5: 148.15 ± 6.27 vs. 114.80 ± 6.76 nmol/mg protein × 8 h, *p* < 0.05) (Figure 3F). Therefore, NZO cells showed a more pronounced response to E2 as a stimulant compared to the NMRI controls. In comparison to non-preincubated cells, HGP reduction was more pronounced after E2 stimulation in cells of the NZO mice (1.6 to 1.9 fold) than in the NMRI controls (1.1 to 1.4 fold). Nevertheless, the utilization could not be further decreased with insulin and E2 co-stimulation. Thus, the overall NZO HGP profiles were nearly similar towards the non-preincubated setting (Figure 3C,F). It was demonstrated that HGP was modulated by E2 in both strains. While co-stimulation with E2 and insulin further reduced the HGP at both time points, the potentiating effect of insulin was not present in the cells of the NZO mice. Since pregnancy-induced cells showed different responses to the added stimulants, further ex vivo experiments were conducted.

### 2.4. E2 Effect on Glucose Uptake and Glycogen Content in Primary Hepatocytes

**Figure 4:**
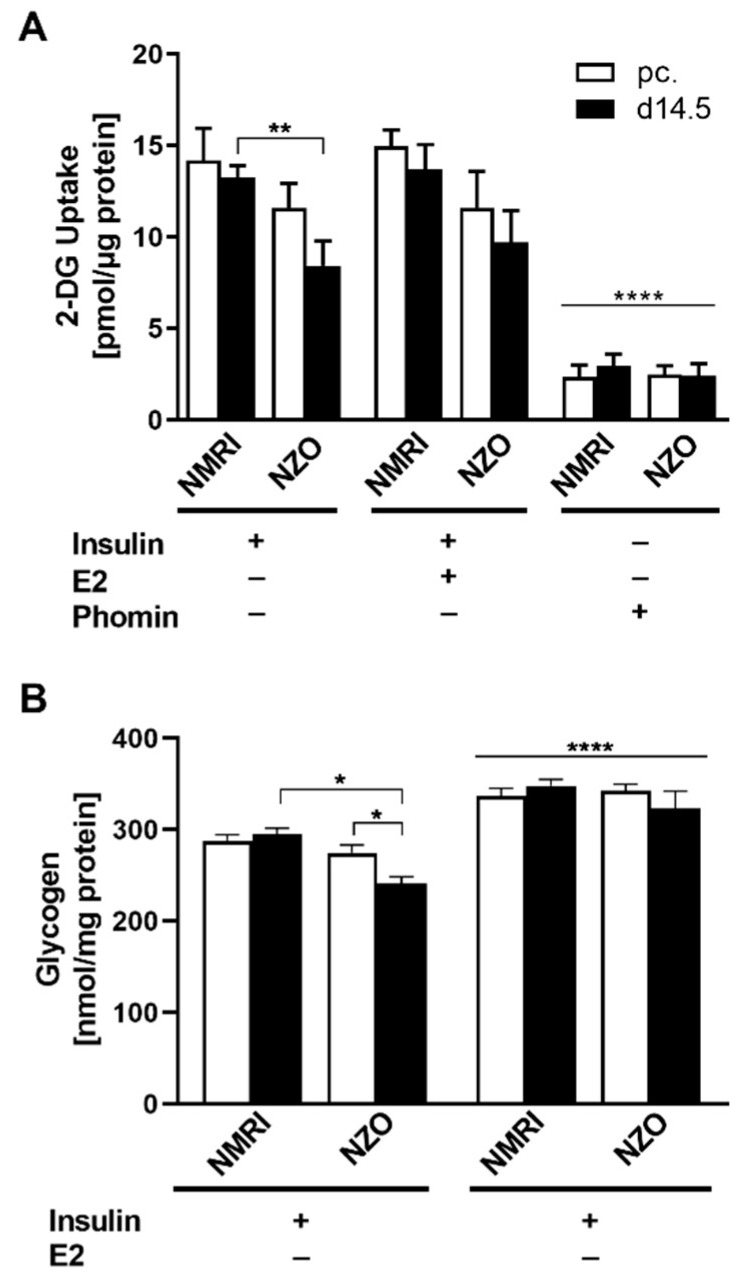
Impact of E2 on glucose uptake and glycogen in primary hepatocytes. (**A**) 2-DG uptake with insulin and E2 co-stimulation or Phomin control and (**B**) glycogen content with insulin preincubation and E2 stimulation in cultured primary hepatocytes of NMRI and NZO mice at time points preconceptional (white bars) and d14.5 (black bars). Data are presented as means ± SEM ((**A**) n = 5–6, (**B**) n = 5 animals per group). * *p* < 0.05, ** *p* < 0.01, multiple comparison tests results between groups: + I vs. + Phomin: **** *p* < 0.0001, + I vs. + I + E2: **** *p* < 0.0001.

Subsequently, HGU and hepatic glycogen content were investigated under ex vivo conditions. After insulin stimulation, 2-deoxy-glucose (2-DG) uptake was significantly reduced at d14.5 in NZO primary hepatocytes compared to NMRI controls (NZO vs. NMRI: 8.43 ± 1.35 vs. 13.26 ± 0.63 pmol/μg protein, *p* < 0.01) (Figure 4A). Co-stimulation with E2 had no effect on 2-DG uptake. In order to prove the absent effect of E2 stimulation, cells were treated with Phomin as an assay control, which resulted in a distinct decrease in 2-DG uptake compared to insulin stimulation. On the contrary, an E2 mediated effect on glycogen content was observed (Figure 4B). After insulin stimulation, NZO cells exhibited a decreased glycogen content at time point d14.5 compared to preconceptional (240.94 ± 7.40 vs. 274.50 ± 8.79 nmol/mg protein, *p* < 0.05). While no difference was observed preconceptionally, after insulin stimulation, the glycogen content in NZO cells was significantly reduced compared to NMRI controls at d14.5 (NZO vs. NMRI: 240.94 ± 7.40 vs. 294.94 ± 6.68 nmol/mg protein, *p* < 0.05). The co-stimulation with E2 and insulin induced a significant increment in glycogen content in both strains preconceptionally as well as during gestation compared to the insulin-stimulated control.

### 2.5. Modulatory Effects of E2 on cAMP and AKT Signaling

**Figure 5:**
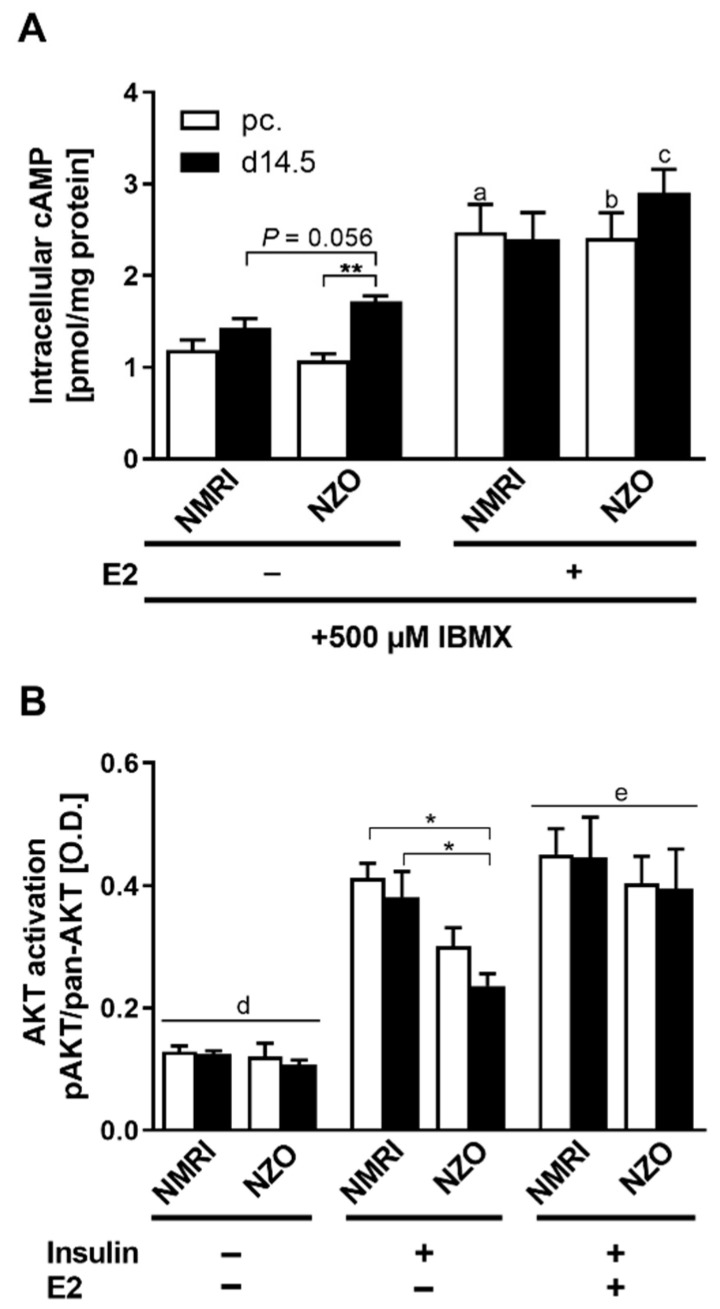
E2 signaling in primary hepatocytes. (**A**) Intracellular cAMP content with E2 stimulation and (**B**) AKT activation (pAKT/pan-AKT ratio) with insulin and E2 stimulation in cultured primary hepatocytes of NMRI and NZO mice at time points preconceptional (white bars) and d14.5 (black bars). Data are presented as means ± SEM ((**A**) n = 5, (**B**) n = 4–6 animals per group). * *p* < 0.05, ** *p* < 0.01, (**A**) NMRI pc. vs. NMRI pc. + E2: a *p* < 0.05, NZO pc. vs. NZO pc. + E2: b *p* < 0.05, NZO d14.5 vs. NZO d14.5 + E2: c *p* = 0.055, (**B**) multiple comparison tests results between groups: + I vs. *w*/*o* I: d *p* < 0.0001, + I vs. + I + E2: e *p* < 0.01.

Next, parts of the regulatory signaling of hepatic glucose metabolism were examined by investigating cAMP and AKT activation. Intracellular cAMP content was examined in primary hepatocytes of both strains (Figure 5A). Preconceptionally, cAMP content was significantly increased in NMRI cells after E2 stimulation compared to unstimulated NMRI controls (2.47 ± 0.30 vs. 1.19 ± 0.11 pmol/mg protein, *p* < 0.05). Unstimulated NZO primary cells already showed significantly increased cAMP content at time point d14.5 compared to preconceptional (1.72 ± 0.06 vs. 1.07 ± 0.08 pmol/mg protein, *p* < 0.01). Additionally, there was a trend to increased cAMP content compared to NMRI cells at d14.5. After the E2 stimulation, NZO cells exhibited higher cAMP content preconceptionally (2.40 ± 0.28 vs. 1.07 ± 0.08 pmol/mg protein, *p* < 0.05) and at d14.5 (2.91 ± 0.25 vs. 1.72 ± 0.06 pmol/mg protein, *p* = 0.055) than their unstimulated counterparts. The AKT activation was measured as the ratio of phosphorylated protein to pan-AKT (Figure S1) after an initial insulin stimulus to induce phosphorylation (Figure 5B). Primary hepatocytes of the NZO mice showed significantly reduced AKT activation at both time points compared to NMRI controls (NZO vs. NMRI, preconceptional: 0.30 ± 0.03 vs. 0.41 ± 0.02, *p* < 0.05; NZO vs. NMRI, d14.5: 0.24 ± 0.02 vs. 0.38 ± 0.04 O.D., *p* < 0.05). The co-stimulation with E2 significantly increased AKT activation in both strains and equalised the decreased activation of the NZO hepatocytes, which abolished the difference in AKT activation between cells of both strains.

## 3. Discussion

In this study, adaptive processes to increased metabolic demands during pregnancy, insulin and glucagon secretion profiles, HGP and HGU, and glycogen content were investigated. Moreover, with AKT activation as well as intracellular cAMP content, parts of the metabolic signaling cascades were investigated. AKT links the activation of the insulin receptor to its downstream suppressive effect on gluconeogenesis and the latter mediates the activation of protein kinase A (PKA), thus acting on glycogenolysis and gluconeogenesis. The study was conducted in prediabetic NZO mice and compared to the metabolically healthy NMRI control strain. We could show that E2 has protective effects on glucose metabolism, both preconceptionally and at d14.5 in prediabetic NZO mice. These findings show that E2 improves the adverse metabolic status in the NZO strain through an increase of insulin secretion. Moreover, E2 improves HGP, HGU, and glycogen content in primary NZO hepatocytes. However, this effect is not sufficient to fully restore the altered glucose metabolism in NZO mice, although it compensates for the increased hepatic IR. Thus, E2 protects against metabolic deterioration and stabilizes GDM progression in NZO mice.

The NZO strain showed elevated plasma E2 levels during gestation. In general, increased E2 levels due to additional placental production are required to sustain pregnancy [33]. There is consensus on the metabolic influence of the hormone on human and rodent metabolism and protective effects of E2 play a decisive role in the prevention of metabolic diseases [34]. A meta-analysis by Ding et al. confirmed a sex-dependent aetiology of T2DM. However, elevated plasma E2 levels were connected to T2DM risk, as adipose tissue-derived E2 was highest in men and postmenopausal women with diabetes [35]. Notably, NZO mice displayed elevated E2 levels even preconceptionally, probably due to the larger size of their ovaries and their follicle numbers compared to other mouse strains [36]. Furthermore, the elevated E2 levels may be caused by decreased levels of luteinizing hormone (LH), commonly seen in certain types of polycystic ovary syndrome (PCOS), for which NZO mice have been described as a model [37,38].

Static incubations with E2 in combination with 20 mM glucose improved GSIS in isolated NZO islets. Increased plasma levels and the enhancement of insulin secretion implicate that E2 functions as a protective, extrapancreatic modulator during gestation in NZO mice. This may explain the enhanced in vivo insulin secretion observed in oral glucose tolerance tests (OGTT) in pregnant NZO mice which was not detectable in ex vivo experiments in which islets were perifused with 20 mM glucose [32]. The protective effect of E2 on the development of diabetes has already been shown in female NZO mice by ovariectomy and the subsequent aggravation of IR and enhancement of β-cell loss [31]. Another study showed that supplementation with E2 in ovariectomized C57BL/6J mice resulted in improved glycemia, glucose intolerance, insulinemia, and insulin secretion [39]. Here, we demonstrated for the first time that E2 may prevent the exacerbation of GDM in prediabetic NZO mice by improving insulin secretion. However, it should be noted that there are probably other modulators besides E2, as additional pregnancy-associated hormones or cytokines may also be involved in improving in vivo insulin responsiveness [32]. Nonetheless, these findings are in line with the improved insulin secretion mediated by E2, already demonstrated ex vivo in mice and humans [40,41,42]. It has also been previously shown that subcutaneous injection of higher physiological doses of E2 increased insulin secretion above levels seen in untreated control rats and were comparable to the increased islet responses seen in pregnant animals [43]. The stimulation of insulin secretion could occur via the ERβ through activation of the guanylyl cyclase A (GC-A) receptor, as previously shown in mice [44]. Furthermore, the insulinotropic effect might also be mediated via the G protein-coupled estrogen receptor (GPER/GPR30) through the epidermal growth factor receptor (EGFR) and extracellular signal-regulated kinase (ERK) [45,46].

Faure et al. observed that E2 treatment reversed the ovariectomy-induced increase in plasma glucagon levels in rats [47]. By stimulation with 20 mM glucose, E2 exhibited no inhibiting effect on glucagon secretion in islets of both strains, suggesting that E2 is not involved in the improvement of elevated glucagon plasma levels during pregnancy in NZO mice [32]. However, it should be mentioned that incubation with 100 nM E2 in combination with 20 mM glucose resulted in increased glucagon secretion in the NZO model. Nevertheless, this difference is most likely related to the high variability in glucagon secretion at 20 mM glucose and thus cannot be considered an actual effect. These findings contrast with observations in which islets of male GPR30^(+/+)^ mice respond to E2 at 20 mM glucose with a decrease in glucagon secretion [45]. However, our results are consistent with other data, where an effect on glucagon secretion by E2 in islets of female NMRI mice was found to be present at low but absent at high glucose concentrations [40]. Thus, the lack of response to glucagon secretion may occur due to an inadequate E2 efficacy at high glucose concentrations.

In early pregnancy, the liver provides precursors of steroid synthesis, while in late pregnancy it supplies glucose to meet the high metabolic demands of the mother and fetus. Thus, liver metabolism and reproduction are closely linked [48]. Although the role of the liver in IR induced by E2-deficiency is still under discussion, data from euglycemic-hyperinsulinemic clamps indicate that IR in female *ERα* KO mice is fully mediated by insufficient restriction of HGP [49]. NZO mice showed increased HGP, a suitable indicator of altered glucose homeostasis and, in addition to IR, a characteristic feature of prediabetes [50]. The physiological mild increase in IR during pregnancy was still recognisable. In contrast, sufficient E2 levels revealed the protective potential on glucose metabolism by suppressing HGP. Remarkably, the response to E2 stimulation was more pronounced in NZO mice compared to NMRI controls indicating a possible compensatory effect. Thus, despite being insulin resistant, NZO mice showed a higher sensitivity to E2 than the controls. This is in line with data demonstrating decreased gluconeogenesis and glycogenolysis after E2 administration in rats as well as in humans [51,52,53]. Thus, increased insulin sensitivity of the liver is modulated by E2 through suppressing gluconeogenesis and promoting glycogen acquisition. The gluconeogenic effect is regulated by hepatic Forkhead box protein O1 (FOXO1) signaling, since the deletion of this transcription factor diminished E2-mediated improvement in glucose homeostasis [54]. These data indicate that the FOXO1 signaling pathway is required for the suppressive effect mediated on HGP by E2. In addition, this could result in the NZO strain not developing hyperglycemia despite an IGT. While overexpression of ERα further improved insulin and E2 induced phosphorylation of FOXO1, it inhibits hepatic gluconeogenesis [54].

Since ERs may localize with the plasma membrane, ligand binding elicits activation of the PI3K/AKT pathway. Moreover, mice lacking the major AKT isoform PKBβ generally develop diabetes-like phenotypes with impaired HGU and insufficient lowering of HGP [55,56]. NZO mice displayed reduced AKT activation, whereas E2 stimulation mediated increased AKT activation. Even in male mice lacking the aromatase gene essential for E2 synthesis, IGT was observed, but insulin and E2 administration could significantly enhance AKT activation. Furthermore, in male *ERα* KO mice, insulin failed to induce hepatic AKT [57,58]. This indicates that the beneficial effects of E2 are not limited to females, although they are more pronounced due to much higher levels, and that functioning E2 signaling is required for unrestricted glucose metabolism. However, in livers of C57BL/6 mice on HFD, increased basal AKT phosphorylation was observed, albeit reduced insulin sensitivity [59]. E2 also shows an ameliorative effect of AKT phosphorylation in the NZO mouse and thus the potential to improve peripheral hepatic IR.

Detection of HGU found no specific E2 effect in either NZO mice or in NMRI. However, insulin induced whole-body glucose uptake has been reported to be reduced in ovariectomized mice and successfully reversed by E2 [60]. This is in line with the concomitantly observed reduced HGP, which taken together leads to an improved glucose homeostasis in the liver.

Diabetes has been linked to an elevated hepatic cAMP content in rats [61,62]. This is in line with our data, which revealed elevated cAMP levels in hepatocytes of the prediabetic NZO mice. Matsumoto et al. observed that the deletion of FOXO1 impaired cAMP-induced hepatic glycogenolysis and gluconeogenesis in primary hepatocytes [63]. Therefore, the involvement of FOXO1 indicates a shared HGP regulation by cAMP and insulin. This is supported by data which revealed the involvement of FOXO1 in insulin-mediated inhibition of cAMP-induced HGP [64,65]. This illustrates the link in HGP regulation of downstream insulin signaling via FOXO1 with cAMP signaling. The demonstrated inducing effect of E2 on intracellular cAMP is contradictory at first glance, since E2 administration lowers HGP. However, it was observed that the increase in cAMP synthesis is directly responsive to E2 [66,67]. Zucchetti et al. further demonstrated that increasing cAMP levels could be induced independently of AKT [68,69]. This observed increase in cAMP can be explained through the E2 effect on GPER, which processes extra-nuclear signaling [70,71]. It was shown that similar to ER-deficiency, *GPER-1* KO mice exhibit IGT and an insulin secretion defect [45,72]. Therefore, the increased cAMP content at d14.5 in NZO mice is consistent with the highest observed plasma E2 levels.

Besides gluconeogenesis, glycogenolysis is a fundamental part of glucose utilization. Achard and Laybutt showed decreased glycogen synthesis in palmitate induced HepG2 cells and in C57BL/6 derived insulin resistant hepatocytes [73]. Reduced glycogen content was observed in NZO mice, which may be an indicator of IR. Irimia et al. already showed that, in glycogen synthase (GS), KO mice increased IR with the concomitant decrease in AKT phosphorylation [74]. Besides the liver, E2 stimulation restored glycogen also in the rodent uterus [75,76]. Glycogen synthesis is dependent of the AKT-mediated glycogen synthase kinase-3α (GSK3α) inactivation, which otherwise impaired the synthesis through inhibiting GS [77]. Therefore, the observed increased AKT activation via E2 might even be a marker for an increment in hepatic glycogen content. Additionally, the reduced HGP after E2 treatment could directly affect the glycogen storages, indicated by a strong correspondence between glucose-6-phosphate (G6P) gluconeogenic substrate, and hepatic glycogen synthesis [78]. Since hepatic glycogen breakdown and storage are crucial for the glycemic control of blood glucose, and E2 exerts a stimulatory effect on its storage, this may indicate why prediabetic NZO mice do not exhibit hyperglycaemia despite showing IGT [32]. As the HGP supressing effect was most pronounced in NZO mice at d14.5, this could be directly related to an E2-mediated increase in hepatic glycogen content.

## 4. Conclusions

In summary, NZO mice show important characteristics of a GDM subtype which expresses IGT before the onset and during gestation. This defect is accompanied by impaired insulin secretion. The aim of this work was to elucidate the cause of improvement of those parameters during gestation in NZO mice. Based on our data shown here, we conclude that E2 acts beneficially on insulin secretion, contributing to the characteristic improvement of GSIS in the NZO phenotype during gestation. Furthermore, an ameliorating effect on hepatic glucose metabolism during gestation was observed. This effect was more pronounced in the islets of Langerhans than in NZO hepatocytes, since the effects on the liver alone are not sufficient to explain the improved whole-body insulin sensitivity during pregnancy. However, our data suggest that an increase of E2 levels during gestation controls a compensatory process that protects against metabolic deterioration and stabilizes GDM progression. Despite the tumour-promoting long-term effects on the female reproductive organs, E2 is an interesting modulator whose effect on glucose metabolism in other tissues such as muscle and fat should be further investigated.

## 5. Materials and Methods

### 5.1. Animals

All procedures were performed under permits from the ethics committee of the Lower Saxony State Office for Consumer Protection and Food Safety (Oldenburg, Germany; ethics approval number: 33.19-42502-04-17/2462; internal IDs (05.15) TSB TU BS and (05.19) TSB TU BS). The NZO (NZO/HIBomDife) and the NMRI (NMRI/RjHan) control strain were used for this study. Due to its ordinary physiological adaptation towards pregnancy and as it has a known robust β-cell physiology, the NMRI strain was chosen as a metabolically healthy control [32,79]. Mice were housed in an air-conditioned room at 21 ± 1 °C with a lighting period comprised of a 12:12 h light-dark cycle (lights on at 06:30 a.m.). Animals had ad libitum access to water and food and were fed a standard chow diet (1328 P, Altromin, Lage, Germany) containing 11% fat, 24% protein, and 65% carbohydrates with a total metabolizable energy of 13.5 kJ/g. At the age of about seven weeks, female NZO and NMRI mice were mated overnight and pregnancy was confirmed by the presence of copulatory plugs the following morning. This day was denoted as 0.5 days’ post coitum and mice were studied at d14.5 of gestation at the age of about 9–10 weeks. The preconceptionally examined control group was the same age as the pregnant one. These animals were used for all performed tissue isolation, perfusion and plasma-related experiments. Animals were sacrificed by cervical dislocation after isoflurane anaesthesia (Isofluran CP 1 mL/mL; CP-Pharma, Burgdorf, Germamy) according to the manufacturer’s instruction. Depending on whether plasma measurements or experiments with primary islets of Langerhans and hepatocytes were performed, the number of animals per group ranged from 5–10. The exact numbers are referenced in the figure legends.

### 5.2. Chemicals

17β-estradiol, Cytochalasin B (Phomin), Collagenase Type IV, Collagenase P (Roche) and Dexamethasone were obtained from Sigma-Aldrich (Steinheim, Germany). William’s Medium E cell culture media was obtained from PAN Biotech (Aidenbach, Germany) and FCS was obtained from Gibco (Life Technologies, Darmstadt, Germany). The RIPA Lysis Buffer was purchased from Thermo Scientific (Waltham, USA) and insulin (Insuman Basal) was purchased from Sanofi (Frankfurt, Germany). All other reagents were obtained from Merck (Darmstadt, Germany), Carl Roth (Karlsruhe, Germany) or PanReac AppliChem (Darmstadt, Germany).

### 5.3. Measurement of Plasma E2

Whole blood was collected from 6 h food deprived animals via the vena cava or the heart. Plasma was obtained by centrifugation (21,000× *g*, 2 min, 4 °C), and samples were stored at −80 °C until further measurement using 17β-estradiol ELISA (abcam, Cambridge, UK) according to the manufacturer’s instruction.

### 5.4. Islet Isolation

Islets of Langerhans were isolated by the collagenase digestion technique (Collagenase P; 0.5 mg/mL) and hand-picked under the stereomicroscope in a HEPES-buffered Krebs-Ringer medium (115 mM NaCl, 4.7 mM KCl, 2.6 mM CaCl_2_, 1.2 mM KH_2_PO_4_, 1.2 mM MgSO_4_, 20 mM NaHCO_3_, 10 mM HEPES, and 2 mg/mL BSA) saturated with 95% O_2_ and 5% CO_2_ containing 5 mM glucose (for details see Grupe et al. [32]).

### 5.5. Ex Vivo Stimulation and Hormone Secretion in Islets of Langerhans

For each experimental set, 10 freshly isolated islets were preincubated for 1 h in Krebs-Ringer medium containing 5 mM glucose. This was followed by a 1 h incubation with HEPES-buffered Krebs-Ringer medium containing 5 or 20 mM glucose in combination with 10, 100, or 1000 nM E2. The medium was collected and stored at −20 °C until further analysis. Hormone concentrations were determined by Glucagon ELISA (Mercodia, Uppsala, Sweden), and Rat Insulin ELISA (Mercodia, Uppsala, Sweden) according to manufacturer’s instruction.

### 5.6. Hepatocyte Isolation and Cell Culture

Hepatocytes were isolated from liver of NZO and NMRI control mice by collagenase digestion technique using a modification of the perfusion method after Baltrusch et al. [80] and studied in short-term primary culture. In short, the peritoneal cavity was opened and a catheter flatly inserted in the portal vein and fixed. The in situ perfusion was executed with an Reglo Analog peristaltic pump (Ismatec, Wertheim, Germany) under continuous flow and a digestion process was performed at 37 °C for 8 to 10 min with Collagenase Type IV. The vena cava was cut to avoid pressure increase within the liver. When the liver structure was disrupted, the capsule including the gall bladder was removed and the gathered cells were kept on ice. The isolated cells were filtered and purified and afterwards suspended in supplemented William’s Medium E (5% FCS, 1% penicillin-streptomycin, 5 mM glutamine (2.5%), 0.5% insulin, 0.1 µM dexamethasone (0.01%) and 11.11 mM glucose). Cell viability was tested by trypan blue exclusion and thereafter cells were seeded on collagen-coated plastic cell culture dishes (Sarstedt Nümbrecht, Germany) at the density required in the single experiment and incubated in a humidified atmosphere at 37 °C, 95% O_2,_ and 5% CO_2_. 0.5 × 10^6^ cells per well were seeded on 6-well plates for glucose production, AKT activation, glycogen, and intracellular cAMP content. For glucose uptake, 5000 cells per well on 96-well plates were seeded. After 3 to 4 h attachment phase, non-adherent cells were removed and fresh supplemented William’s Medium E (5% FCS, 1% penicillin-streptomycin, 5 mM glutamine (2.5%), 0.5% insulin, 1 µM dexamethasone (0.01%), and 11.11 mM glucose) was added.

### 5.7. Ex Vivo Stimulation of Primary Hepatocytes

Hepatocytes were cultured overnight (8 h) with indicated stimulant in serum-free William’s Medium E and dexamethasone before experiments were conducted. For stimulation the following concentrations were used: 1 nM insulin in glucose production assay, and 100 nM insulin in all other experiments. The E2 stimulus was 100 nM. All experiments were performed within 48 h after plating.

### 5.8. Glucose Production

Primary hepatocytes were washed with PBS and incubated in a supplemented glucose-free DMEM starvation medium (1% HEPES, 2% BSA, 44 mM NaHCO_3_, 2.5% glutamine and 1% penicillin-streptomycin) for 8 h. Hepatocytes were then provided with glucose production supplemented DMEM medium (without phenol red, 2 mM sodium pyruvate and 20 mM sodium L-lactate as gluconeogenic substrates) and with the indicated stimulant. Then the glucose was measured by fluorometric hexokinase glucose assay (abcam) according to the manufacturer’s instruction over 8 h. Assay results were normalized for protein content using a BCA protein assay kit (Sigma-Aldrich).

### 5.9. Glucose Uptake

Primary hepatocytes were starved in a Krebs-Ringer-Phosphate-HEPES (KRPH) buffer (20 mM HEPES, 5 mM KH_2_PO_4_, 1 mM MgSO_4_, 1 mM CaCl_2_, 136 mM NaCl, 4.7 mM KCl and 2% BSA) for 40 min and supplemented with the indicated stimulant. Afterwards, the medium was removed and a fluorometric 2-DG glucose uptake assay (abcam, Cambridge, UK) was performed according to the manufacturer’s instruction. Briefly, cells were stimulated with 100 nM insulin and the indicated stimulant for 20 min and then 2-DG was added and cells were incubated for an additional 20 min at 37 °C. The hepatocytes were washed with PBS to remove exogenous 2-DG and to terminate reaction. Afterwards cells were lysed, freeze-thawed and heated at 85 °C for 20 min and the assay was conducted. Assay results were normalized for protein content using a BCA protein assay kit (Sigma-Aldrich, Steinheim, Germany).

### 5.10. Glycogen Content

Primary hepatocytes were washed with phosphate-buffered saline (PBS) three times and then incubated with William’s Medium E without glucose and pyruvate for 2 h to deplete glycogen. Afterwards, cells were incubated with William’s Medium E plus 25 mM glucose and 100 nM insulin supplemented with the indicated stimulant for an additional 2 h. The cells were then washed with cold saline, incubated with 30% KOH for 30 min, and scraped and incubated again at 95 °C for 20 min. The lysate was then cooled and mixed with absolute ethanol and kept at 4 °C overnight. The cellular glycogen was precipitated by centrifugation (15,000× *g*, 10 min, 4 °C). The supernatant was discarded, and the pellet (glycogen fraction) was suspended in water. The glycogen content was determined with a fluorometric glucoamylase assay kit (abcam, Cambridge, UK) according to the manufacturer’s instruction. Assay results were normalized for protein content using a BCA protein assay kit (Sigma-Aldrich, Steinheim, Germany).

### 5.11. Measurement of Intracellular cAMP

Primary hepatocytes were treated with 500 µM 3-isobutyl-1-methylxanthine (IBMX) and the indicated stimulant 30 min prior further processing to accumulate cAMP in cells. The cells were washed with PBS and treated with 0.1 M HCl for 10 min at room temperature. Successful lysis was ensured by microscopic inspection. To get rid of cellular debris, centrifugation (2800× *g*, 3 min) was performed. Supernatants were assayed immediately according to the method of Johanns et al. [81]. Therefore, the content was determined using the direct cAMP ELISA kit (Enzo Life Sciences, Farmingdale, USA) according to the manufacturer’s instruction in the acetylated format to increase assay sensitivity. Assay results were normalized for protein content using a BCA protein assay kit (Sigma-Aldrich Steinheim, Germany).

### 5.12. Measurement of AKT Activation

Primary hepatocytes were stimulated with the indicated stimulant for 3 h and then 100 nM insulin and stimulant were added 10 min prior procession in serum free William’s Medium E. Lysates of primary hepatocytes were prepared in a modified RIPA buffer (150 mM NaCl, 50 mM Tris, pH 7.6, 1% Triton X-100, 0.5% Sodium deoxycholate and 0.1% SDS) supplemented with cOmplete^TM^ protease inhibitor (Roche, Mannheim, Germany) and a protein phosphatase inhibitor set (Sigma-Aldrich, Steinheim, Germany) described by Titchenell et al. [82]. Successful lysis was ensured by microscopic inspection. Supernatants were extracted from cellular debris following centrifugation (15,000× *g*, 10 min). The AKT activation was then assessed with Phospho-AKT(pSer473)/pan-AKT ELISA kit (Sigma-Aldrich, Steinheim, Germany) according to the manufacturer’s instruction and protein content was assessed using a BCA protein assay kit (Sigma-Aldrich, Steinheim, Germany).

### 5.13. Shared Control Groups

Since the conducted ex vivo experiments with primary hepatocytes in this study were part of a series of experiments with different stimulants, the same experimental protocols were used in accordance with the 3R principles [83]. In addition, according to Törnqvist et al. [84], to reduce the number of animals, several stimulation experiments were performed in parallel in order to require only one control group. Where applicable, the control groups of glucose production, glucose uptake and glycogen content, as well as intracellular cAMP content and AKT activation, were shared and may be used by our research group in other publications. The corresponding author for these publications is in all cases Dr. Stephan Scherneck.

### 5.14. Data Handling and Statistics

Statistical analysis and graphical presentation were performed by Prism 8 and Prism 9 (GraphPad, La Jolla, USA) software. All data were expressed as means ± standard error of the mean (SEM). Outliers were identified and removed using the ROUT method. This method was used for the following parameters: Figure 2A–D: insulin and glucagon secretion after static incubation. For data analysis, non-parametric statistics were applied. Statistical analysis was performed using a Mann-Whitney U test for the comparison of two groups and a Kruskal-Wallis H test followed by a Dunn’s multiple-comparison test was applied for the comparison of several groups. For further multiple-group comparisons of different stimulation groups, a two-way ANOVA was used. Differences were considered significant at *p* < 0.05. *p* values were indicated as * *p* < 0.05, ** *p* < 0.01, *** *p* < 0.001 and **** *p* < 0.0001.

## Data Availability

All data presented in this study are available on request from the corresponding author.

## Supplementary Materials

The following supporting information can be downloaded at: www.mdpi.com/article/10.3390/ijms23126693/s1.
