# Supplementary material for: Estradiol (E2) Improves Glucose-Stimulated Insulin Secretion and Stabilizes GDM Progression in a Prediabetic Mouse Model
Source: Int J Mol Sci. 2022 Jun 15;23(12):6693. doi: 10.3390/ijms23126693 (PMC9223537; doi:10.3390/ijms23126693)
Supplement: Supplementary file 1 [file ijms-23-06693-s001.zip › ijms-1758024-supplementary.pdf]

International Journal of
Molecular Sciences

MDPI

Supplementary Materials: Estradiol (E2) Improves Glucose-Stimulated Insulin Secretion and Stabilizes GDM Progression in a Prediabetic Mouse Model

Moritz Liebmann †, Melissa Asuaje Pfeifer †, Katharina Grupe and Stephan Scherneck

Institute of Pharmacology, Toxicology and Clinical Pharmacy, Technische Universität Braunschweig, Mendelssohnstraße 1, D-38106 Braunschweig, Germany; m.liebmann@tu-braunschweig.de (M.L.); melissa.asuaje-pfeifer@tu-braunschweig.de (M.A.P.); k.grupe@tu-braunschweig.de (K.G.)

* Correspondence: s.scherneck@tu-braunschweig.de; Tel.: +49-531-391-8440

† These authors contributed equally to this work.

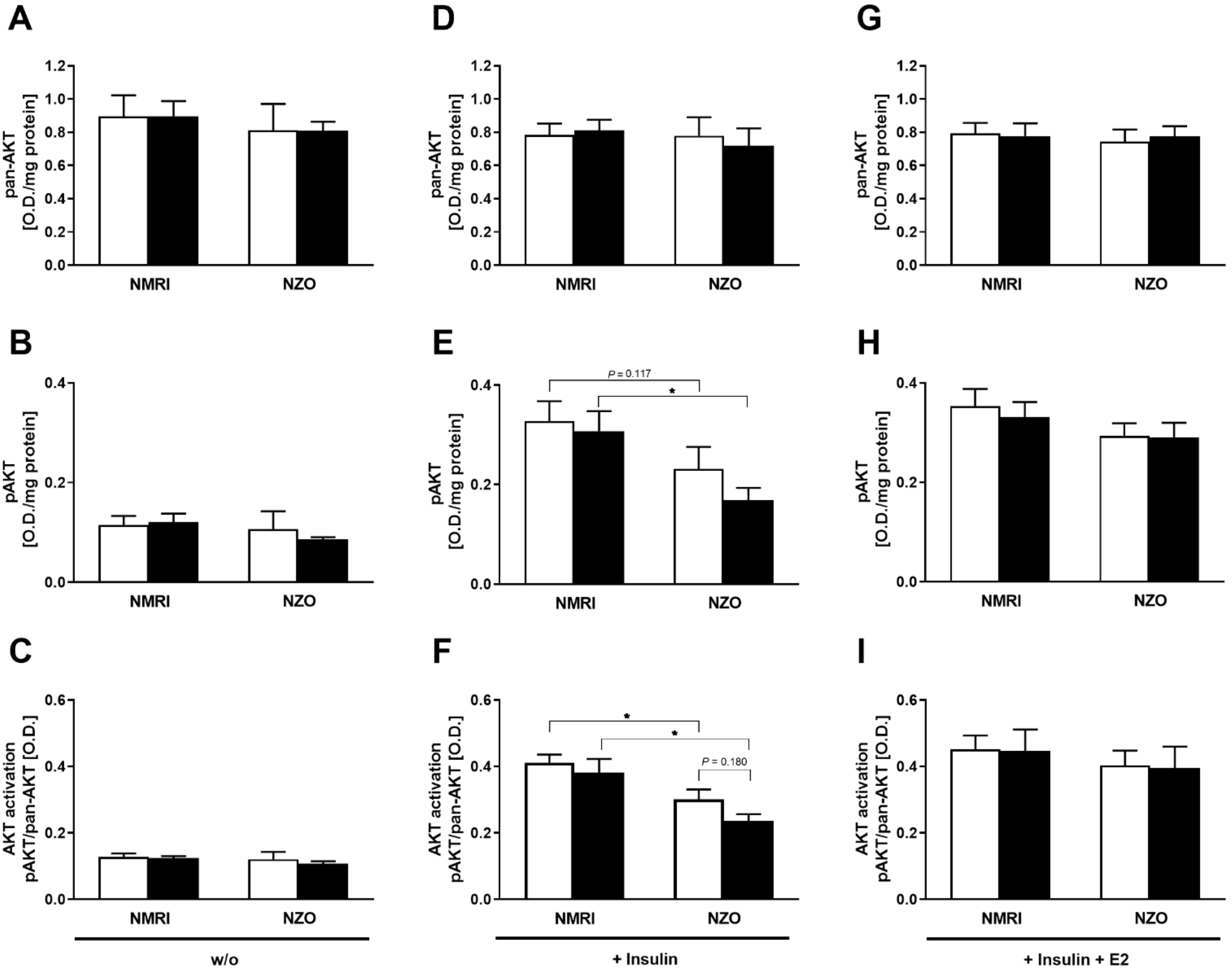

Figure S1. AKT signaling in primary hepatocytes after E2 stimulation. (**A**, **D**, **G**) Pan-AKT, (**B**, **E**, **H**), phospho-AKT and (**C**, **F**, **I**) the derived pAKT/pan AKT-ratio with insulin and E2 stimulation in primary hepatocytes of NZO and NMRI (controls) mice at time points preconceptional (white bars) and d14.5 (black bars). Data are presented as means ± SEM (n = 4-6 animals per group). *p<0.05.
